# Heat shock-optimized CRISPR/Cas9 system for visible clonal analysis and mutant generation in *Drosophila*

**DOI:** 10.1093/g3journal/jkaf236

**Published:** 2025-10-07

**Authors:** Yi Lin, Xiaolei Ye, Ling Zeng, Yuting Luo, Ying Feng, Yan Zhang

**Affiliations:** State Key Laboratory of Eye Health, Eye Hospital, Wenzhou Medical University, Wenzhou, Zhejiang 325027, China; Drosophila Resources and Technology Platform, Eye Hospital, Wenzhou Medical University, Wenzhou, Zhejiang 325027, China; State Key Laboratory of Eye Health, Eye Hospital, Wenzhou Medical University, Wenzhou, Zhejiang 325027, China; Drosophila Resources and Technology Platform, Eye Hospital, Wenzhou Medical University, Wenzhou, Zhejiang 325027, China; State Key Laboratory of Eye Health, Eye Hospital, Wenzhou Medical University, Wenzhou, Zhejiang 325027, China; Zhejiang Key Laboratory of Key Technologies for Visual Pathway Reconstruction, Eye Hospital, Wenzhou Medical University, Wenzhou, Zhejiang 325027, China; State Key Laboratory of Eye Health, Eye Hospital, Wenzhou Medical University, Wenzhou, Zhejiang 325027, China; Zhejiang Key Laboratory of Key Technologies for Visual Pathway Reconstruction, Eye Hospital, Wenzhou Medical University, Wenzhou, Zhejiang 325027, China; State Key Laboratory of Eye Health, Eye Hospital, Wenzhou Medical University, Wenzhou, Zhejiang 325027, China; State Key Laboratory of Eye Health, Eye Hospital, Wenzhou Medical University, Wenzhou, Zhejiang 325027, China; Zhejiang Key Laboratory of Key Technologies for Visual Pathway Reconstruction, Eye Hospital, Wenzhou Medical University, Wenzhou, Zhejiang 325027, China

**Keywords:** *Drosophila melanogaster*, CRISPR/Cas9, heat shock, clonal analysis, mutant generation

## Abstract

In *Drosophila* genetic studies, clonal analysis such as mosaic and Mosaic Analysis with a Repressible Cell Marker has been widely used to investigate gene function. Recently, the CRISPR/Cas9 system has been established as a powerful tool for efficient mutant generation; however, its application in clonal analysis has been rarely reported. Here, we present a suite of Gal4/UAS-Cas9 binary expression systems that integrate UAS-Cas9 and *multiple-sgRNAs* (*single-guide RNAs*) into a single plasmid. These systems facilitate versatile applications, enabling Gal4-driven direct phenotypic studies, approximate clonal analysis, in vitro cell transfection, and stable mutant generation, among which, the third-generation constructs: *G3a/b* incorporate visible labeling strategies for marking approximate clonal regions. In addition, compared to continuously active drivers, we found that the short-pulse-induced *heat shock*-*Gal4* (*hs-Gal4*) was sufficient to induce high clonal efficiency and generate larger clones. In the germline, short-pulse heat shock is also effective. It reduces residual Cas9 activity in the germline stem cells, thereby minimizing the risk of affecting germline stem cell survival and improving mutant acquisition.

## Introduction

For over a century, *Drosophila melanogaster* has served as a fundamental model organism in genetic and developmental studies, greatly advancing our investigation of gene function, developmental principles, and disease mechanisms (Koon and Chan 2017; Yamaguchi and Yoshida 2018; Mirzoyan et al. 2019; Ogienko et al. 2022; Jeong 2024). Among *Drosophila* clonal analysis system—including mosaic analysis (Xu and Rubin 1993), Mosaic Analysis with a Repressible Cell Marker (MARCM) technique (Lee and Luo 1999), germline clone analysis (Larsson and Rasmuson-Lestander 1998), and other flippase/flippase recognition target (FLP/FRT)-mediated genetic methods—have become essential for genetic studies.

More recently, additional clonal analysis techniques have been developed to study cell lineage formation, such as CoinFLP (Bosch et al. 2015), Bitbow (Li et al. 2021), and lineage filtering (Awasaki et al. 2014). FLP/FRT-mediated clonal analysis is based on the mitotic chromosomal recombination between 2 FRT sites within the same locus and the same direction in both homologous chromosomes (Theodosiou and Xu, 1998). For example, in mosaic analysis, chromosomal translocation is induced to generate mutation clones, and then the clonal regions are labeled by the absence of GFP. These clones are typically surrounded by GFP-positive wild-type or heterozygous genotypic clones, thus providing the internal control regions (Blair 2003). In contrast, the MARCM technique employs Gal80 to suppress *UAS-GFP* expression. Only after a mitotic recombination event exchanges the chromosome arm carrying Gal80 with the arm carrying the mutation via FRT sites, then the GFP expression is released, resulting in mutation clones that coincide with GFP expression. Unlike the mosaic analysis, MARCM labels mutant cells positively via GFP expression, making it particularly suitable for studying cells with complex spatial structures, such as neurons or intestinal cells (Lee and Luo 1999).

In addition, when 2 FRT sites in the same orientation are arranged linearly on the same chromosome, FLP-mediated recombination induces excision of the DNA fragment between them (Golic et al. 1997; Theodosiou and Xu, 1998). This mechanism contributed to the invention of the FLP-out technique (Theodosiou and Xu, 1998). In contrast, 2 inversely oriented FRT sites induce inversion of the intervening fragment (Bosch et al. 2015; Manivannan et al. 2019). We have often used Gal4/UAS and FLP-out systems to drive ectopic gene expression (Zhong and Yedvobnick 2009) or RNA interference (RNAi) (Dietzl et al. 2007; Ni et al. 2008) for large-scale genetic screens. RNAi-based screening has improved efficiency and enabled higher-throughput functional studies, while issues also exist, such as variable RNAi efficiency and off-target effects remain persistent challenges (Kaya-Copur and Schnorrer 2016). However, the more precise gene manipulation at the DNA level, conventional methods often involve complex genetic procedures that are time-consuming and unsuitable for high-throughput studies.

Throughout the history of generating mutants, the methodologies have evolved from early techniques relying on natural mutations (Xu and Rubin 1993), radiation-induced mutagenesis (Chia et al. 1986), chemical mutagenesis (Blumenstiel et al. 2009), transposable element insertion (Caceres et al. 1999), and imprecise P-element excision (Ou, 2013) to more precise and controllable strategies. In recent years, precise genetic engineering has been greatly facilitated by the development and widespread adoption of engineered nucleases, including the zinc-finger nuclease (ZFN) technique (Rebar and Pabo 1994; Bibikova et al. 2002), transcription activator-like effector nuclease (TALEN) system (Wood et al. 2011; Liu et al. 2012), and CRISPR/Cas9 system (Jinek et al. 2012; Gratz et al. 2013; Ghosh et al. 2016). Notably, the CRISPR/Cas9 system has revolutionized the field of biological sciences by offering a highly efficient and versatile platform for genome editing and functional genetic studies.

In *Drosophila*, CRISPR/Cas9 has been widely adopted for targeted gene knockout (Bassett et al. 2013; Xue et al. 2014), knock-in (Bosch et al. 2020; Yu et al. 2021; Avellaneda and Schnorrer 2022), transcriptional regulation (Lin et al. 2015; Han et al. 2022), and chromatin modification (Yu et al. 2013). However, conventional mutagenesis usually requires more steps including mutant generation, genetic mapping, and subsequent recombination with other genetic elements. Moreover, for many homozygous viable genes, clonal analysis remains necessary to clearly discern phenotypes, such as in growth competition assays (Tanaka et al. 2016). To overcome these mutagenesis efficiency issues, several studies have leveraged the Gal4/UAS binary system to drive Cas9 expression directly in specific tissues, enabling the generation of mutant clones in situ and significantly improving the efficiency of phenotypic detection (Xue et al. 2014). A common strategy involves crossing UAS-Cas9 lines with newly established single-guide RNA (“sgRNA”) strains, followed by recombination with various Gal4 drivers. However, this process remains cumbersome and limits the flexibility of Gal4 usage. To streamline these efforts, we developed a suite of all-in-one constructs that integrate UAS-Cas9 with *multiple-sgRNA* expression cassettes. This design simplifies experimental workflows and enhances the scalability of CRISPR/Cas9-based genetic screening. We have successfully validated this system through more than 20 independent transgenic flies, confirming its high efficiency in phenotypic investigation and mutant generation.

Subsequently, we developed the uniform pipeline of CRISPR/Cas9-mediated investigation and knockout strategies. Previously, we found that the Cas9 knockout regions often did not precisely match the Gal4 expression, and resulting clones frequently appeared in random locations, which depended on which Gal4 line was selected. This study demonstrated that our all-in-one system, when driven by eye-specific, wing-specific, and other Gal4 drivers, could induce clear defective phenotypes and was even applicable for lethality and reproductive studies. Our results confirmed previous findings that the Cas9 system often exhibited a low probability of precise coincidence between the Cas9-induced knockout region and the Gal4 expression domain. Among the drivers tested, *en-Gal4* and *hs-Gal4* in the *G3a* system yielded the best spatial concordance, and *hs-Gal4* could generate larger clones for observation.

Once promising phenotypes were identified using our systems, we can employ germline-specific drivers such as *nos-Gal4* to generate stable FRT-mutant lines directly. In our *hs-Gal4* system, *hs-Gal4* usually combines with the FRT site in the chromosome arm, which also performs effectively in producing stable mutants directly with a FRT site. Such a suite of systems provides valuable methodology for more in-depth gene functional studies.

## Materials and methods

### Fly stocks

*G1-dual-sgRNAs(GFP)*, *G1-dual-sgRNAs(H2Av)*, *G1-dual-sgRNAs(CG6236)*, *G1-dual-sgRNAs(nonstop)*, *G1-dual-sgRNAs(dom)*, *G2-dual-sgRNAs(rbo)*, *G3a-dual-sgRNAs(H2Av)*, *G3a-dual-sgRNAs(dlp)*, *G3a-dual-sgRNAs(mad)*, *G3b-dual-sgRNAs(H2Av)*, and *FRT82B-H2Av^Cas9d^* were generated for this study. *ey-Gal4*, *hh-Gal4*, *ap-Gal4*, *en-Gal4*, *ptc-Gal4*, *hs-Gal4*, *UAS-nlsGFP*, (*hs-flp; FRT82B, Ubi-GFP*), (*hs-flp*; *Ay-Gal4, UAS-GFP)*, (*hs-flp; UAS-GFP; Tub-Gal4, FRT82B, Tub-Gal80*), and *H2Av^810^* were obtained or assembled from components of Bloomington *Drosophila* Stock Center (Department of Biology, Indiana University, Bloomington, IN, USA).

### Fly breeding and genotypes

All directly CRISPR/Cas9 global knockout flies were kept at 25 °C. For *G3a hs-Gal4* activation in larvae, flies were heated in a 37 °C water bath twice for 30 min each with a 12 h interval on the second day after egg laying. For *G3b* flies were kept in a 29 °C incubator for 24 h on the second day after egg laying and then changed back to 25 °C until final investigation. The breeding conditions of germline mutagenesis are shown in the last paragraph of results and demonstrated in Fig. 5a, which describes the detailed time points and treatment temperatures.

The following are the fly genotypes in this study:

Figure 2a: *ey-Gal4*/+;*G1-dual-sgRNAs(GFP)/+*

Figure 2a’: *ey-Gal4*/*G1-dual-sgRNAs(H2Av); +/+*

Figure 2a’’: *ey-Gal4*/*G1-dual-sgRNAs(CG6236); +/+*

Figure 2a’’’: *ey-Gal4*/*G1-dual-sgRNAs(not); +/+*

Figure 2a’’’’: *ey-Gal4*/*+*; *G1-dual-sgRNAs(dom)/+*

Figure 2b to b’’: *ey-Gal4*/*+*; *UAS-nlsGFP/+*

Figure 2c to c’’’: *ey-Gal4*/*G1-dual-sgRNAs(H2Av)*; *UAS-nlsGFP/+*

Figure 2d to d’’: *+/+*; *hh-Gal4/G2-dual-sgRNAs(rbo)*

Figure 3a to a’’’: *hs-flp/+ or Y*; *Ay-Gal4,UAS-GFP/G1-dual-sgRNAs(H2Av); +/+*

Figure 3b to b’’’: *ap-Gal4*/*G1-dual-sgRNAs(H2Av)*; *UAS-RFP/+*

Figure 3c to c’’’: *en-Gal4*/*G1-dual-sgRNAs(H2Av)*; *UAS-GFP/+*

Figure 3d to d’’’: *ptc-Gal4*/*G1-dual-sgRNAs(H2Av)*; *UAS-RFP/+*

Figure 3, e to e’’ and f to f’’: *hs-flp/+ or Y;UAS-GFP/G1-dual-sgRNAs(H2Av)*; *Tub-Gal4,FRT82B,Tub-Gal80/FRT82B*

Figure 4, a, a’, and b to b’’’: *hs-Gal4/G3a-dual-sgRNAs(H2Av); Ubi-GFP/+*

Figure 4, c, c’, d, and d’, and Supplementary Fig. 2A to A’’’: *Gal80^ts^/G3b-dual-sgRNAs(H2Av); Tub-Gal4,UAS-GFP/*+

Figure 4, e and g: +/+;+/+ (*W^1118^*)

Figure 4f to f’’’: *hs-Gal4/G3a-dual-sgRNAs(dlp); Ubi-GFP/+*

Figure 4, h and h’, and Supplementary Fig. 3a to b’’: *hs-Gal4/G3a-dual-sgRNAs(mad); Ubi-GFP/+*

Figure 5a: *nos-Gal4*/*G1-dual-sgRNAs(H2Av); gal80^ts^* or *hs-Gal4/G1-dual-sgRNAs(H2Av); +/+*

Figure 5, d and d’: *hs-flp/+ or Y;+/+;FRT82B,Ubi-GFP/FRT82B-H2Av^810^*

Figure 5, e and e’: *hs-flp/+ or Y;+/+;FRT82B,Ubi-GFP/FRT82B-H2Av^Cas9d^*

### Antibodies and immunofluorescence staining

All imaginal wing discs and optic lobes were dissected from *Drosophila* third instar larvae, fixed in 4% fresh paraformaldehyde (PFA, Sigma-Aldrich, V900894) for 20 min at room temperature, and then washed 3 times for 5 min each. The blocking and antibody incubation buffers were PBST (Phosphate Buffered Saline with Triton X-100, 0.1% Triton X-100) with 5% fetal bovine serum. Primary antibodies were used as follows: chicken anti-GFP (1:1,000, Abcam, ab13970), rabbit anti-RFP (1:1,000, Rockland Immunochemicals, 600-401-379), rabbit anti-H2Av (1:400, Active Motif, 39715), mouse anti-Dally-like (Dlp) [1:50, Developmental Studies Hybridoma Bank (DSHB), 13G8], rabbit anti-pMad (1:400, Cell Signaling Technology, 41D10), mouse anti-Flag (1:1,000, Sigma-Aldrich, B3111), rabbit anti-Flag (1:1,000, Abcam, ab1162), and the cell nuclei were stained by DAPI (Sigma-Aldrich, D9542). Fluorescence-conjugated secondary antibodies were obtained from Thermo Fisher Scientific and used at 1:400 dilution: donkey anti-chicken Alexa Fluor 488 (A11039), donkey anti-rabbit Alexa Fluor 488 (A21206), donkey anti-mouse Alexa Fluor 488 (A21202), donkey anti-rabbit Alexa Fluor 546 (A10040), donkey anti-mouse Alexa Fluor 647 (A32787), donkey anti-rabbit Alexa Fluor 647 (A31573). The antifade reagent used for microscope slides was SlowFade Gold Antifade Mountant (Invitrogen, S36937).

### Cell culture

*Drosophila* S2 cells were cultured at 25 °C in Schneider's *Drosophila* Medium (Gibco, 21720024) with 5% fetal bovine serum (Gibco, 10100147). All transfection experiments were carried out using Effectene Transfection Reagent (Vazyme, T101). Each well was seeded with 5 × 10^6 S2 cells, allowing them to grow around 70% to 80% density, then transfected with 300 ng each of *armadillo* (*arm*)*-Gal4* and *G2-dual-sgRNA(H2Av)* plasmids. Cells were fixed and stained for 72 h after transfection.

### Image acquisition

Fluorescence images were obtained on a Carl Zeiss LSM880 with Airyscan platform.

## Results

### The generation of multipurpose CRISPR/Cas9 constructs for *Drosophila* genetic engineering

In *Drosophila* genetic studies, researchers have extensive experience with a wide range of gene manipulation and editing methods, both in vitro and in vivo. For example, we can alternatively employ Cas9 and *sgRNA* tools to perform targeted gene knockouts or inject *sgRNA* plasmids into Cas9 carrier strains to obtain the mutant. However, these procedures often lack flexibility and require preserving more constructs and maintaining numerous fly strains. To address these challenges, we have developed an integrated and robust CRISPR/Cas9-mediated workflow.

**Fig. 1.**
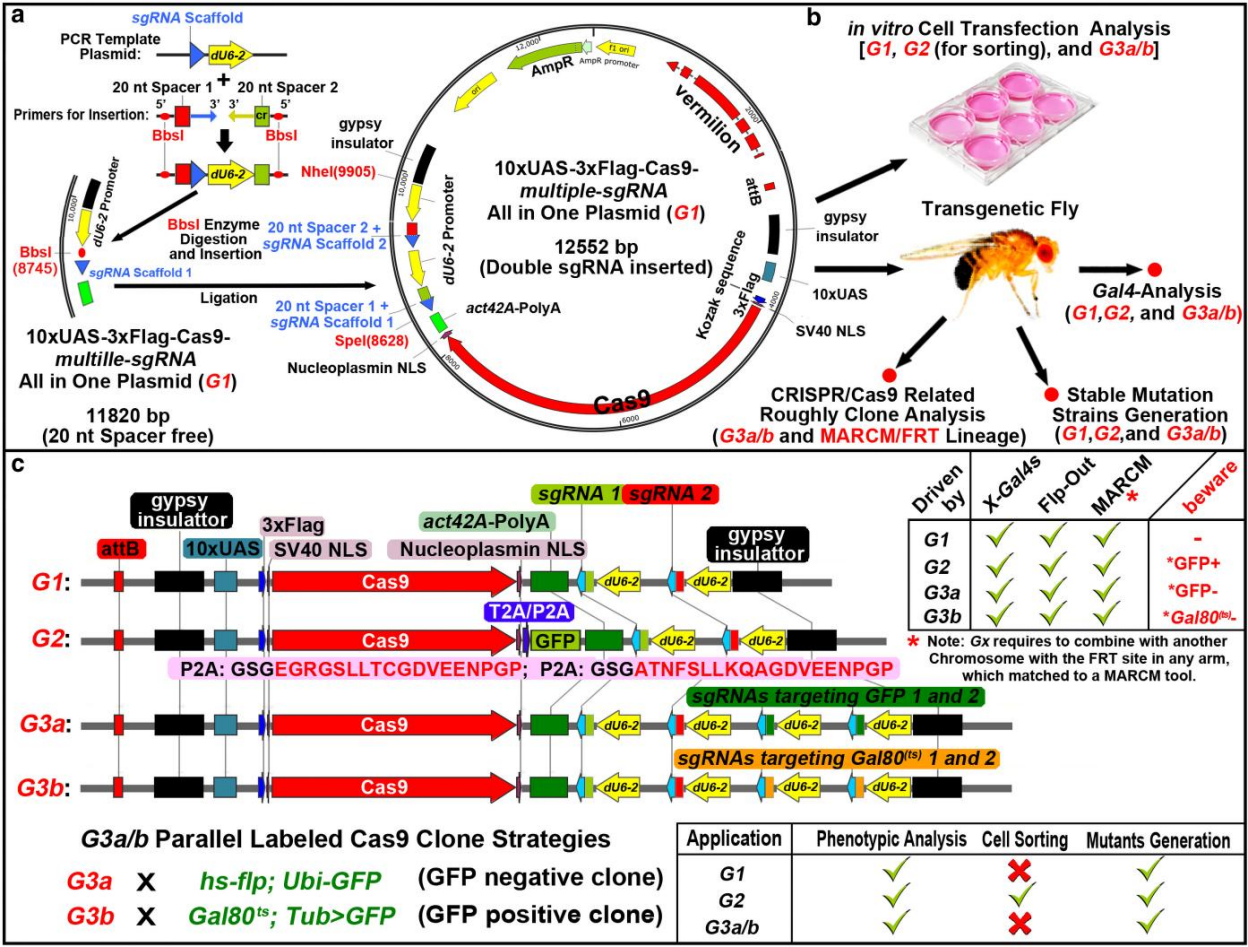
Basic plasmid information and strategic framework. a) The *G1* construct: *UAS-Flag-Cas9-multiple-sgRNA* all-in-one plasmid scaffold and *dual-sgRNA* assembly strategy. A single *sgRNA* can be generated by a pair of complementary oligos by annealing and inserting into the BbsI (8745) cloning site; double *sgRNAs* require insertion of a BbsI-digested PCR product from another template plasmid shown in the upper-left corner of a. Additional *sgRNAs* can be inserted into SpeI (8628) and NheI (9905) unique cloning sites on both sides. b) Multiple applications of use *G1*, *G2*, and *G3a/b*. All generations of these plasmids can be used for transfection into cell lines in vitro, for transgenesis to generate fly strains, and to obtain stable mutations following the protocols introduced later. When planning to use a parallel visible marker, the *G3a* or *G3b* system should be chosen. c) The linearized structures of the different plasmid generations. The upper-right corner of the panel illustrates genetic cross strategies with other tool strains. *G3a/b* can also be crossed with tools like *G1* or *G2*; however, the *sgRNAs* targeting *GFP* or *Gal80^(ts)^* coding sequence may disrupt the experimental designs with them.

The first-generation (*G1*) construct was generated based on the flySAM2.0 plasmid (Han et al. 2022). We replaced dCas9 (deadCas9, catalytically inactive Cas9) with the wild-type Cas9 and then replaced its polyA with the *act42A-*polyA sequence, which is functional in both somatic and germline cells. Additionally, its *sgRNA* scaffold was reverted to the standard form used in the Cas9 system. It encompasses *UAS-Cas9* (with a 3× Flag-tag at the N-terminus) and a BbsI enzyme cloning site for inserting a 20 nt *sgRNA* spacer or the digested PCR fragment for *multiple-sgRNA* insertion. This system is adaptable for the insertion of a single 20 nt *sgRNA* spacer to form a single *sgRNA* (via annealing 2 reverse and complementary oligos and inserting them with overhangs at both ends), double *sgRNAs* generated by inserting a *dual-sgRNA* PCR fragment (Fig. 1a), and 4 or 6 *multiple*-*sgRNAs* utilizing SpeI and NheI restriction enzyme cleavage sites for additional *dual-sgRNA* insertion. The *G1* plasmid architecture and multiple purposes of *G1* to *G3* constructs are illustrated in Fig. 1b, and the linearized structures of *G1*, *G2*, and *G3a/b* are depicted in Fig. 1c. Although multiplex splicing with endogenous *tRNA* processing has been reported for the generation of *multiple*-*sgRNAs* simultaneously (Xie et al. 2015), for single or *dual-sgRNAs*, the conventional tandem strategy is relatively straightforward and efficient. To design *sgRNAs* with minimal off-target effects, we utilized the “Optimal Target Finder” online platform (Gratz et al. 2014). The plasmid possesses an *attB* integration element and a *vermilion* selection marker for eye color selection, facilitating phiC31-mediated transgenesis and screening for positive transgenic flies.

**Fig. 2.**
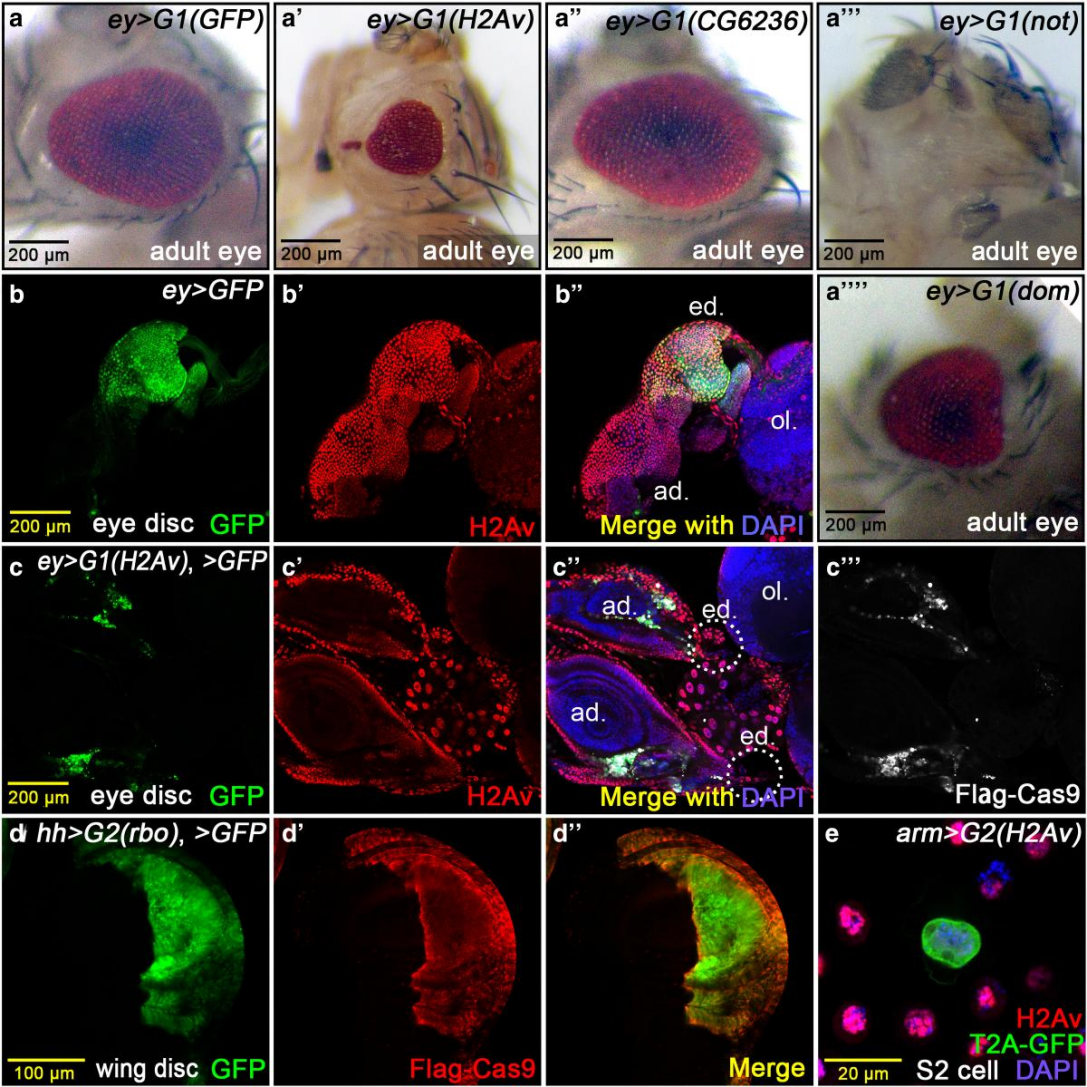
*CRISPR/Cas9-sgRNA* all-in-one constructs perform in vivo and in vitro tasks. *Drosophila* strains carrying the Gal4/UAS binary expression system enable direct drive of CRISPR/Cas9 knockout in tissues and organs, allowing for easy observation of visible phenotypes. When using *ey-Gal4* to drive this system, we found that compared to the control group (a, G1 targeting GFP), the number of ommatidia in the compound eye was significantly reduced in a’ (*G1* targeting *H2Av*). Knocking out the alkaline phosphatase-like gene *CG6236* (a’’) showed no obvious difference from the control, while the *nonstop* group showed complete loss of the eye (a’’’). The *dom* group also showed smaller eyes, but the phenotype was less severe than in the *H2Av* group (a’’’’). The *dom* group also showed shrunken eyes but less severe than the *H2Av* group (a’’’’). b and b’) A third instar larval eye-antennal disc from *ey-Gal4*, *UAS-GFP* controls stained with anti-H2Av antibody. b’’) The merged picture of b and b’. c to c’’’) Global knockout of *H2Av* driven by *ey>G1(H2Av)* system severely affected eye disc (ed.) development (within white dashed lines), while antennal disc (ad) development was unaffected. The optic lobe (ol.) is indicated. d to d’’) Staining of *G2* systems in wing discs, which contain a 2A self-cleaving peptide sequence, confirmed that T2A, P2A, or a combination of both work well for generating GFP markers and functional Cas9. d to d’’) *hh-Gal4 > G2(rbo)* in wing discs, *Gal4*-driven regions were stained via anti-Flag-antibody for the N-terminal tag of Cas9, while T2A-GFP in d’ and merged channels in d’’. e) We also tested the *H2Av* knockout using the *G2* construct in the S2 cell line, which worked acceptably.

The *G2* generation plasmid contains a T2A or P2A sequence (Fig. 1c) for Cas9 and GFP protein split expression, which can express GFP simultaneously with Cas9, facilitating in vivo study (Fig. 2d and d’) or in vitro cell transfection for labeling or flow cytometry sorting (Fig. 2e and Supplementary Fig. 1). *G1* and *G2* transgenic flies can be directly regulated by specific Gal4 drivers for direct phenotypic observation, thereby enabling the assessment of target gene function and facilitating future genetic manipulations.

Since the expression pattern and efficacy of Cas9, when driven by diverse Gal4 drivers, can be variable, identifying the actual editing region is a crucial issue. The antibodies against the target gene should be employed to monitor the knockout performance. Using *H2Av* (*histone H2A variant*) as our model gene, co-staining with an anti-H2Av antibody allowed us to easily distinguish gene knockout regions (where staining is lost). However, due to the lack of antibodies for novel genes of interest, we attempted to resolve this issue by employing parallel labeling strategies. Therefore, we have developed a method based on the GFP absence labeling and the GFP present labeling strategies (utilizing the Gal80^ts^ system to inhibit Gal4 activity, analogous to the principle of MARCM technology). These 2 strategies introduce additional *sgRNAs* targeting the *GFP* or *Gal80^ts^* coding sequence. Consequently, we designated them as CRISPR/Cas9-Mediated Parallel Visible Marked Clonal Analysis, abbreviated as Marked CRISPR/Cas9 Clonal Analysis. The genetic cross schemes for all *G1*, *G2*, and *G3a/b* constructs with their respective Gal4 drivers are illustrated in the upper-right corner of Fig. 1c, and the genotypes of the specific genetic tools used for *G3a/b* crosses are shown in the lower-left corner.

The complete genetic descriptions of *G1* to *G3* are as follows: *G1*, *UAS-Flag-Cas9, dU6-(multiple)-sgRNA(s)[target gene]*; *G2*, *UAS-Flag-Cas9-(T/P2A)-GFP, dU6-(multiple)-sgRNA(s)[target gene]*; *G3a*, *UAS-Flag-Cas9, dU6-dual-sgRNAs(GFP), dU6-(multiple)-sgRNA(s)[target gene]*; *G3b*, *UAS-Flag-Cas9, dU6-dual-sgRNAs[Gal80^(ts)^], dU6-(multiple)-sgRNA(s)[target gene]*. In the subsequent paragraph, the corresponding contents are abbreviated, such as *G1(H2Av)* in main paragraphs and figures, and *Gx-(multiple/dual)-sgRNA(s)[target gene]*, such as *G1-dual-sgRNAs(H2Av)* for genotypic description in materials and methods. The primers for PCR and Sanger sequencing used in plasmid creation, the sequence of PCR template for creating *dual-sgRNAs*, and other relevant detailed information are listed in the supplementary document (Supplementary Doc. 1). The *UAS-Flag-Cas9-dual-sgRNAs* (*G1*) example sequence (used 20Ns and 20Ms indicate 2 spacer sequences) is provided in a supplementary*.dna* file.

### Gal4/UAS-Cas9 binary system applicable in adult eyes, larval organs, and S2 cells

In our initial tests, we used certain *Gal4s*, such as *ey-Gal4* and *en-Gal4*, to investigate the direct phenotypic defects achievable through this system. As depicted in Fig. 2, a to a’’, we employed the CRISPR/Cas9 knockout system directly driven by *ey-Gal4* to scrutinize the phenotypic consequences of knocking out genes such as *H2Av*, *CG6236*, *not* (*nonstop*), and *domino* (*dom*) in the adult eye. We determined that this system reliably produces evident mutant phenotypes. *H2Av* knockout caused developmental defects in the adult eye, resulting in a significantly reduced eye size (Fig. 2a’). Knockout of *CG6236* (Fig. 2a’’), an alkaline phosphatase-like gene associated with the Toll-Imd immune pathway, exhibited no significant phenotypic difference in the eye compared to the *sgRNAs* targeting GFP control group (Fig. 2a, as Cas9 system control). *not* is an indispensable functional gene encoding an H2B single-deubiquitination factor, which influences the elongation of RNA transcription. The absence of *non* results in the death of third instar larvae, and *non* knockout in the eye leads to extremely small or completely obliterated eye in adult (Fig. 2a’’’). The gene *dom* encodes a component of the complex responsible for the replacement of the variant histone H2Av with canonical H2A on chromatin (Kusch et al. 2004). In its absence, the *dom* mutant phenotype is similar to that of the *H2Av* defect. As *dom* is genetically upstream of H2Av, and H2Av may also be subject to additional redundant regulation or have broader functions, the deficiency of *dom* is less severe than H2Av loss (Fig. 2a’’’’).

We employed the *G1* all-in-one plasmid for global knockout in the eye-antennal imaginal disc of third instar larvae. Compared to the control group (Fig. 2b to b’’), Fig. 2, c and c’, demonstrates that a complete organ loss results from growth arrest in eye discs. The *G2* generation plasmids are primarily designed for the convenience of in vitro cell transfection and sorting by flow cytometry. The *G2* plasmid incorporates a T2A sequence, which enables the co-expression of 2 independent proteins via a ribosomal skipping mechanism that results in internal cleavage during translation. Besides T2A, we can also opt for P2A or link them together to form a tandem P2A-T2A domain (Daniels et al. 2014). Figure 2, d and d’, illustrates the *CRISPR/Cas9-dual-sgRNA* system utilizing *hh-Gal4* to drive Cas9 with *dual-sgRNAs* targeting the *rbo*, also named *stambha A* (*stmA*) gene in the imaginal wing disc, demonstrating that the expression pattern of Flag-Cas9 is highly consistent with that of T2A-GFP. This strategy allows for effective gene knockout within labeled regions by GFP monitoring. Particularly when applied to in vitro cell culturing, we utilized this system to knock out *H2Av* in S2 cells. As demonstrated in Fig. 2e, it yielded an acceptable knockout efficiency and was suitable for subsequent cytometry sorting work.

### Different Gal4 drivers resulted in distinct regional patterns

In this study, we generated a series of all-in-one constructs targeting specific genes, injected them to create transgenic flies, and then examined the developmental, reproductive, and survival outcomes by crossing these flies with distinct Gal4 drivers. Although the CRISPR/Cas9 system is highly effective and convenient, its editing regions are often not consistently and stably aligned with the expression pattern of the Gal4 drivers.

**Fig. 3.**
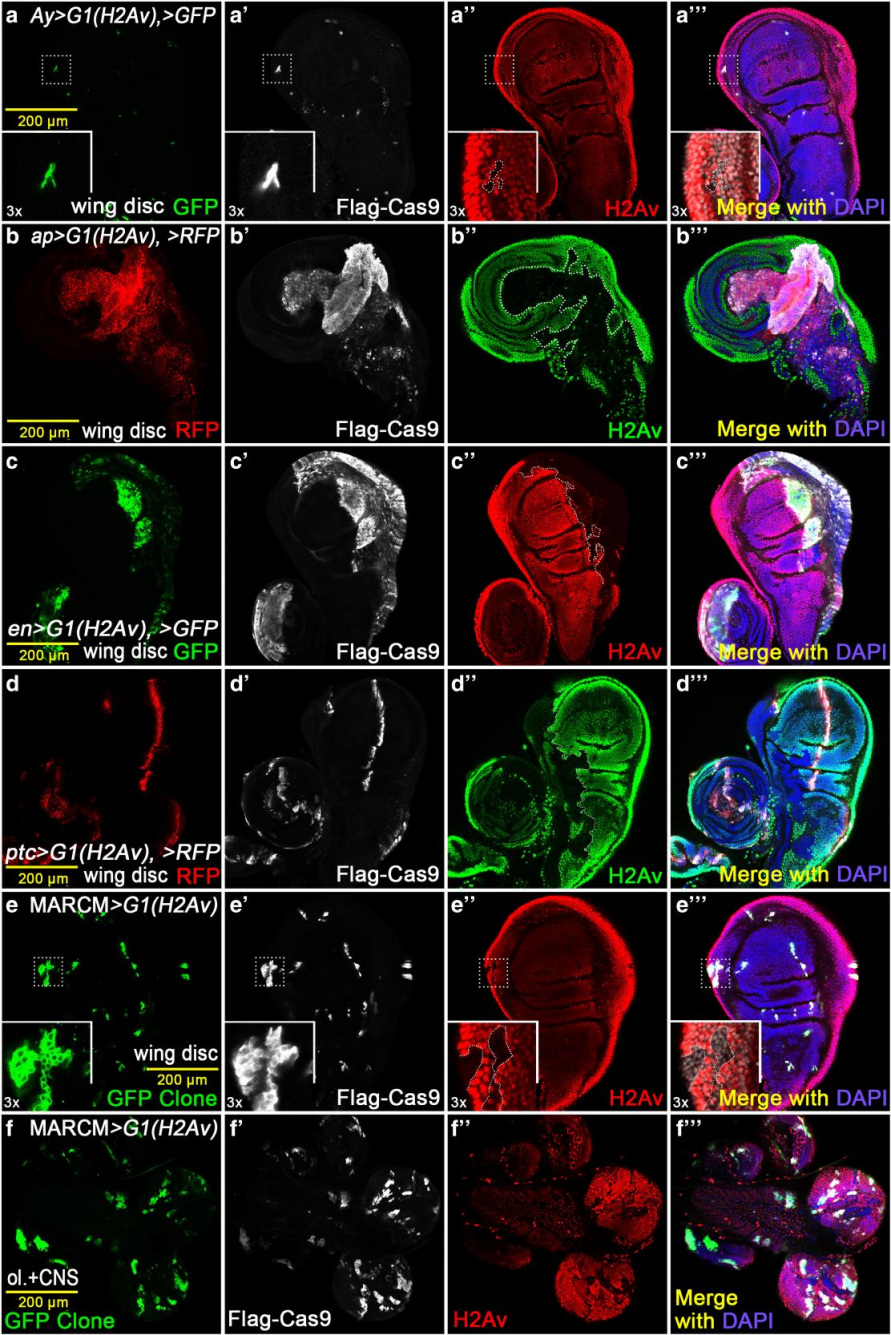
Diverse *Gal4* drivers show various regional knockout effects. Gal4 drivers exhibit significant differences in their guidance of the UAS-Cas9 system’s gene knockout effects. a to a’’’) Knockout using the *Ay > G1(H2Av)* FLP-out system resulted in a low probability of generating large clones. The clones are extremely small but show good co-localization between Cas9 expression and the knockout region. b to b’’’) The knockout effect of *ap-Gal4* driving *G1(H2Av)*. The RFP expression regions driven by *ap-Gal4* are largely consistent with Cas9 expression, but the expression intensities are not perfectly correlated. c to c’’’) *en-Gal4*-driven *G1(H2Av)* results. The knockout region primarily occurs within the en-driven domain, indicating that *en-Gal4* is well-suited for imaginal wing disc studies. d to d’’’) *ptc-Gal4-*driven *G1(H2Av)*. The RFP expression regions are largely consistent with Cas9, but the clone position is shifted entirely to the anterior side of the wing disc. e to e’’’) The MARCM system driving empty-FRT clones. Cas9 expression closely follows clone formation, but the clone sizes remain small. f to f’’’) The MARCM *> G1* system creating clones in the central nervous system and optic lobe, showing better clonal and knockout efficiency than in the wing disc.

Substantial differences in knockout efficiency and regional editing patterns were observed when using specific Gal4 drivers with the *G1* system. When using the *Ay-Gal4* driver with the FLP-out system to generate CRISPR/Cas9 clones, we found that the system has a low probability to produce the exceedingly small clones (Fig. 3a to a’’’). The *ap-Gal4* group exhibited an easy and robust ability to drive Cas9 expression; however, the *H2Av* knockout regions did not fully align with the Gal4 expression domains. Furthermore, although the overall affected areas were broadly consistent, unexpected knockout events were also identified (Fig. 3b to b’’’). In contrast, *en-Gal4* driving produced the most efficient and optimal knockout effect, with the editing regions being almost perfectly consistent with its expression domains (Fig. 3c to c’’’). The situation of the *ptc-Gal4* group was quite peculiar, as it possesses a certain ability to drive knockout, but all knockout regions shifted to the anterior part of the wing disc. This may reflect the early-stage expression pattern of *ptc* in the wing disc (Fig. 3d to d’’’). We also utilized the MARCM system to generate wild-type clones expressing Cas9 (using an FRT chromosome without mutation in the chromosome arm). In this setup, Cas9 expression was driven within the clones, which were marked by GFP. It showed that the knockout regions were confined to the clones, but the knockout efficiency and the size of the clones were unsatisfactory (Fig. 3e to e’’’). However, the MARCM method performed more effectively in the optic lobe (Fig. 3f to f’’’).

### CRISPR/Cas9-mediated visible clonal analysis facilitates genetic studies

The efficacy of CRISPR/Cas9-mediated editing is augmented by its variable efficiency in individual cells, which may depend not only on Cas9 and *sgRNA* expression levels but also on the chromatin state within the local cells. Direct markers of Cas9 expression, such as Flag-tagged Cas9 or T2A/P2A-GFP, are not sufficient to reliably delineate the regions where functional gene editing has occurred.

**Fig. 4.**
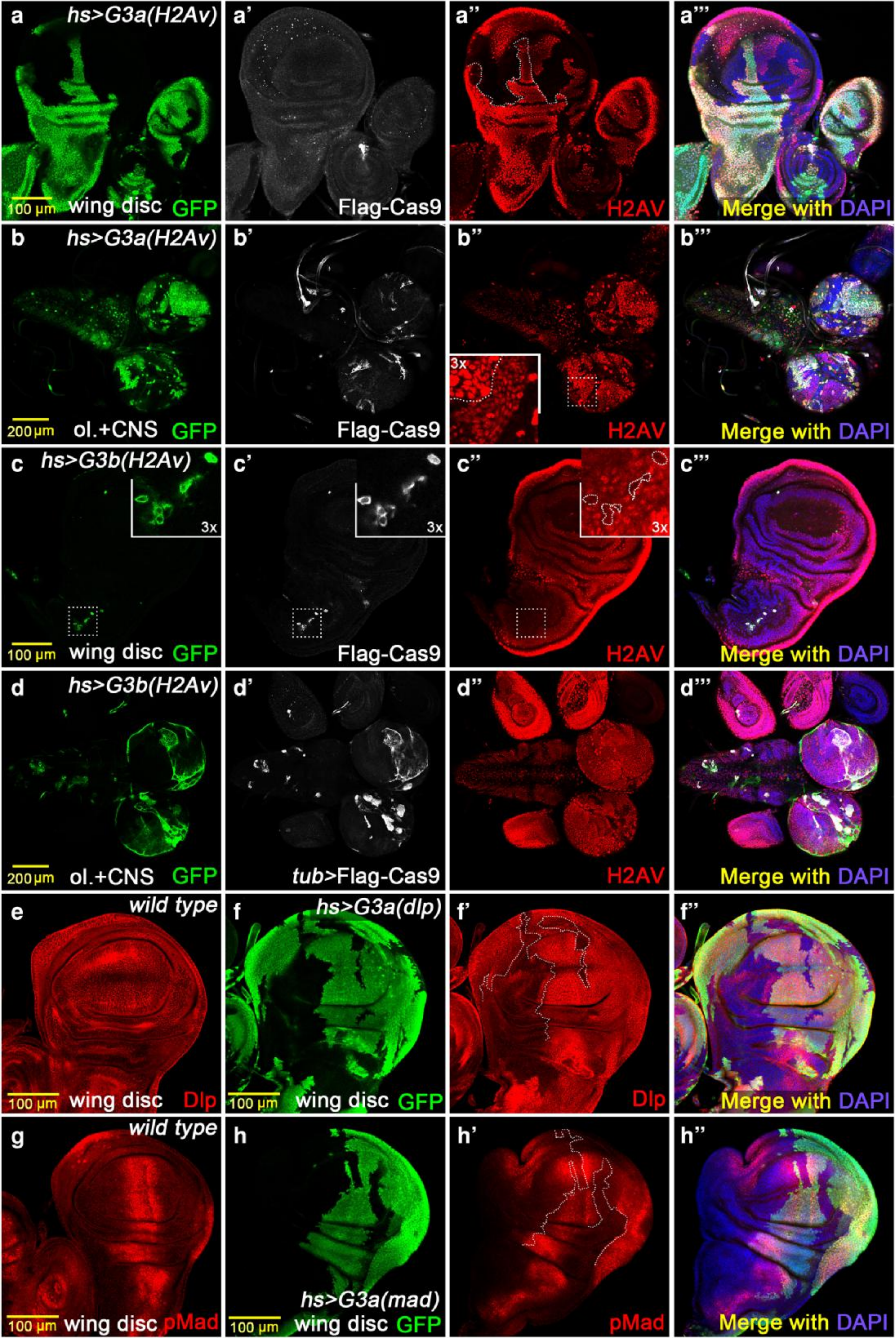
*G3a* and *G3b* parallel visible marker clonal strategies. We designed 2 parallel visible marker strategies for UAS-Cas9 clones, *G3a* and *G3b*, to address the lack of suitable antibodies for detecting knockout regions. The *G3a* system incorporates *sgRNAs* targeting the GFP coding sequence. *hs-Gal4* driving generates larger clones and efficient knockout, although mono-allelic knockout (affecting only one allele) also occurs within the clones (a to a’’’). The *G3a* system performs well in the wing disc. It also performs well in the optic lobe (b to b’’’). The *G3b* system utilizes knockout of the Gal4 inhibitor Gal80^ts^ to release GFP expression, creating GFP-positive zones that indicate potential knockout regions. This method produces smaller clones and weaker knockout efficiency in imaginal discs (c to c’’’) but performs better in the optic lobe (d to d’’’), although still not as effectively as *G3a*. e) Dlp immunofluorescence staining in wild-type wing disc. To verify broad applicability, we knocked out the *dlp* gene using the *G3a* system, which also showed good knockout efficiency (f to f’’). g) Anti-pMad staining in wild-type wing disc. We also used *G3a* to knock out the *mad* gene. h to h’’) pMad staining demonstrates that knockout efficiency is higher at the disc periphery in well-developed samples, while the pouch center is partially affected.

Consequently, we devised a strategy incorporating a parallel editing marker within the same system to effectively monitor CRISPR/Cas9 editing efficiency. Our findings indicated that while parallel labeling strategies may not fully meet all anticipated requirements, both the clone size and labeling specificity were superior to conventional tag immunostaining or T2A/P2A-based GFP reporting. Hence, we designed 2 strategies, designated *G3a* and *G3b*. *G3a* incorporated an additional pair of *sgRNAs* targeting the *GFP* coding sequence. In a background of ubiquitous GFP expression, GFP-negative clones most accurately indicate the knockout efficiency and approximate the location of the target mutation. After comparing the methods using different drivers, we discovered that using *hs-Gal4* could yield larger clones and excellent gene knockout efficiency. The results illustrate that biallelic knockout occurred in broad regions, while mono-allelic knockouts also existed in some areas (Fig. 4a to a’’’). This *G3a* clonal labeling strategy is similar to that used in traditional mosaic analysis. The *G3a* system also operated effectively in the optic lobe (Fig. 4b to b’’’). We further found that even trace amounts of Cas9 expression were sufficient for promoting gene knockout. For example, the *hs-Gal4-*driven Cas9 transient expression was almost undetectable by anti-Flag antibodies in later third instar larval stages (Fig. 4a’ and b’).

For the *G3b* strategy, we designed another pair of *sgRNAs* targeting the *Gal80^ts^* coding sequence. To conditionally activate *Gal4* expression, temperature-sensitive *Gal80^ts^* was introduced into the system. The progeny were exposed to 29 °C for 24 h to allow CRISPR/Cas9 to knock out *Gal80^ts^* in random regions and were then shifted to a lower temperature of 25 °C. In this way, only cells in which *Gal80^ts^* was knocked out could persistently express Cas9, thereby increasing the probability of target gene knockout in those cells. The results indicated that positive-labeling strategy performed poorly in the imaginal wing disc, resulting in small clones and poor efficiency of *H2Av* knockout (Fig. 4c to c’’’). However, the *G3b* system shows optimally in the optic lobe (Fig. 4d to d’’’) and the third instar larval wing disc primary trachea (Supplementary Fig. 2).

To further verify the general applicability of the *G3a* system, we also designed *G3a* constructs targeting *dlp* (*dally-like*, a cell surface heparan sulfate proteoglycan) and *mad* (*mother against dpp*, a transcription factor in the Dpp/BMP signaling pathway) and generated corresponding transgenic flies. Similarly, we observed that the *G3a* system could produce large *dlp* mutant clones in the imaginal wing disc, and the *dlp* knockout regions were largely consistent with GFP-negative areas (Fig. 4f to f’’). In the *mad* clonal analysis groups, we found that *mad* mutant clones in the wing disc growth center may severely impacted disc development (Supplementary Fig. 3). This resulted in a high incidence of wing disc loss in larvae. This survivor bias meant that only discs without, or with only partial knockout in the critical region, could be analyzed. However, at the boundary of wing discs, *mad* knockouts were easily observed (Fig. 4h to h’’ and Supplementary Fig. 3).

The CRISPR/Cas9 clonal technique is most applicable with the antibodies against the target gene, which makes it clear for accurate mutation region. Otherwise, the *G3a* system provides an approximate clonal region labeling method. Subsequently, a stable mutant generation may be necessary for precise investigation.

### Generation of mutants using the CRISPR/Cas9 binary system

Finally, the stable mutants can also be obtained using this system. Here, we selected the pupal homozygous lethal gene *H2Av* and the homozygous viable gene *CG6236*, among others, for validation. This system can be driven by germline-specific drivers, such as *nos-Gal4* and *vas-Gal4*, or by the general driver *hs-Gal4*. In this study, we observed the knockout efficiency in both F1 male and female offspring. We found that the probability of successful knockout depended on the gene characteristics, with genes essential for GSC (Germline Stem Cell) development being more difficult to knockout. In addition, generating long-distance deletion was also more challenging. We attempted to generate a large fragment deletion, such as deletion of ∼7.6 kb gene sequence in the *dom*, after 2 rounds of trying with each involving over 100 single-fly crosses, resulting in a failure to obtain the full-length knockout.

The knockout detail procedure is as follows: F1 progeny carrying *hs-Gal4* was heat-shocked for 2 h per day for 3 consecutive days and then returned to a 25 °C incubator. Subsequently, individual F1 males were crossed with 2 to 3 female balancers. Individual F1 females were grouped (3 virgins per group) and crossed with male balancers. The first batch of hatched F2 offspring underwent mixed pool genotyping PCR (about 30 individuals per pool, priority with females). If a positive PCR band was found, then single F2 males fly cross with balancers respectively, which come from the same well in the first or second batch. After the balancer females laid enough eggs, take a single male fly PCR to confirm the mutation wells and then select the offspring to establish stable mutant lines.

For the *nos-Gal4* driver, we used a temperature-sensitive Gal80^ts^ system to achieve conditional repression of Gal4 activity. After crossing, F1 offspring were placed at 29 °C for 3 d to induce knockout in the GSCs and turn back breeding at 25 °C. Then the F1 males and females were crossed with balancers, and the protocol of identification of positive F2 individuals was the same as that used in the heat shock system. We also confirmed that continuously maintaining flies at 29 °C was also effective for generating mutants. Typically, 16 single-fly crosses were set up in parallel; if there failure in getting a positive well, an additional 16 single-fly crosses needed to be set up. Generally, for a positive F1 pool, the F2 positive well could be identified with 16 to 32 individuals using single-fly PCR. The fly stocks used for transgenic docking and mapping are listed in Supplementary Doc. 1.

**Fig. 5.**
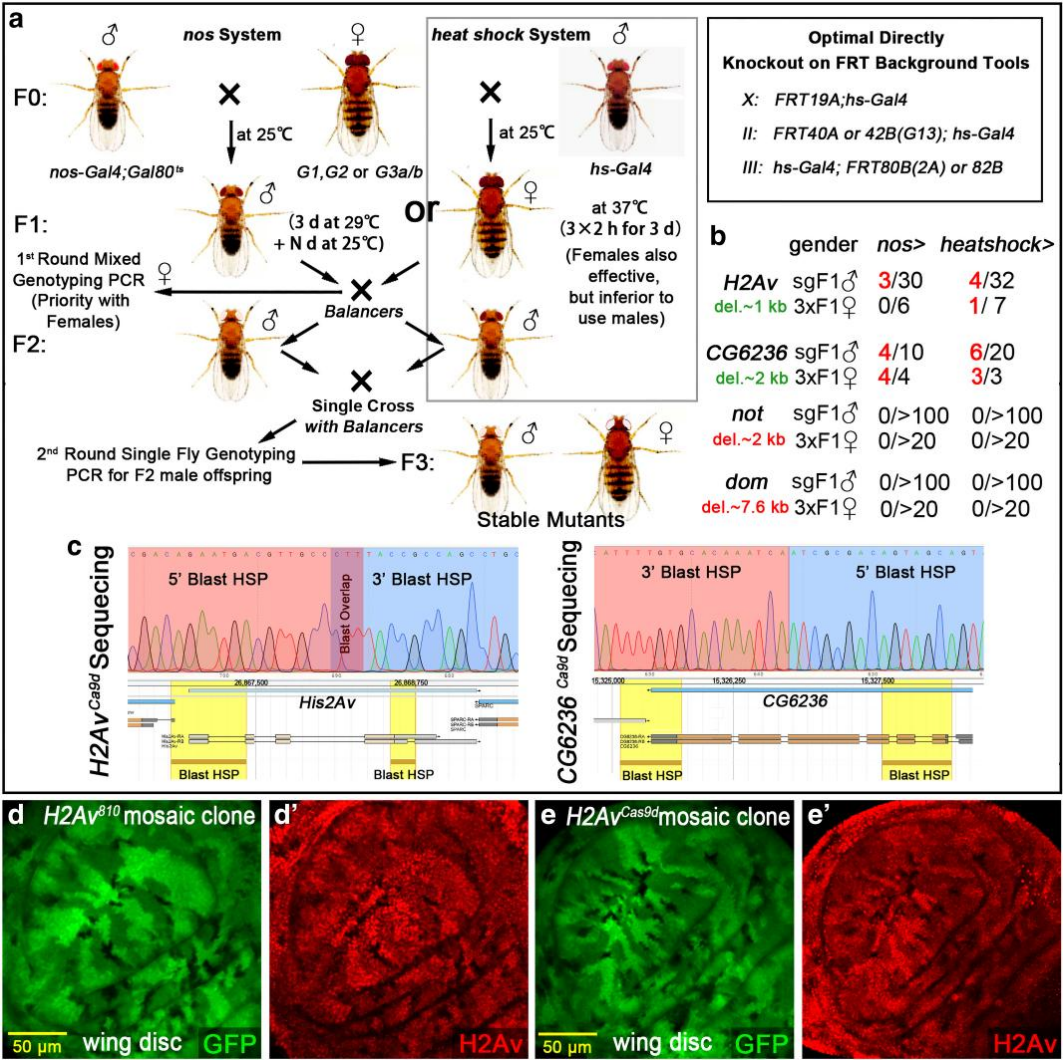
Germline knockout strategies for generating stable mutants. All transgenic flies (*G1* to *G3*) can be used to generate knockouts in germline stem cells, enabling the production of stable mutant strains. The schematic diagram (a) illustrates 2 strategies using *nos-Gal4* or *hs-Gal4* to drive Cas9 expression in the germline. For the genes tested, *nos-Gal4* and *hs-Gal4* drivers showed no major difference in knockout efficiency. The success of knockout depended on the target fragment size and the essentiality of the gene. b) *H2Av* and *CG6236* were relatively easy to knock out. Stable mutants were readily obtained in the offspring of both male and female F1 flies, regardless of the method used. However, genes like not and *dom* were not easily knocked out. Attempts often resulted in short deletions around a single *sgRNA* site, while obtaining full-length deletions to create null alleles was difficult. This semi-precise knockout can be sufficient for strong loss-of-function phenotypes. c) The deleted regions are consistent with the 2 *sgRNA* target sites. We used traditional mosaic clone analysis to characterize the *H2Av^810^* (d and d’) and *H2Av^Cas9d^* (e and e’) mutants. This confirms that the heat shock-driven Cas9 system can directly generate FRT-based mutant alleles.

There is an optimal strategy to generate mutants directly on an FRT chromosome. We generated a suite of *hs-Gal4* with FRT-combined tools for X, II, and III chromosome mutagenesis (Fig. 5, upper-right box). This setup allows the mutation to be directly recombined with the FRT site. The overall genetic cross scheme and temperature conditions are illustrated in Fig. 5a, and the corresponding results and mutant acquisition rates are shown in Fig. 5b.

Short-term heat shock could minimize the risk of excessive biallelic knockout in germline stem cells (GSCs), which may help improve the efficiency of generating specific mutants. Thus, the heat shock-optimized CRISPR/Cas9 system represents an alternative method for manipulating genes that affect germline stem cell maintenance. Genomic sequencing results of the Cas9-induced mutations in *H2Av* and *CG6236* are shown in Fig. 5c. The probability of obtaining mutants using the *nos-* or *hs-Gal4*-driven CRISPR/Cas9 system in positive F1 males is approximately between 10% to 30% (by genotyping PCR results), which is significantly higher than that achieved by the direct injection method (injecting *sgRNA* plasmids into Cas9 carriers). In our lab, the direct injection method consistently yields a positive rate often less than 5%, and the F2 male mutation rate was evaluated by *H2Av* and *CG6236* knockout tests. In the *nos* system, they averaged 10% and 40% (Fig. 5b), respectively. In contrast, the heat shock system achieved the rates of 12.5% and 30% (Fig. 5b), respectively.

The loss of *H2Av* (dark regions) restricted cell proliferation and development in the wing disc pouch, leading to small and fragmented clones. Clone sizes were determined by comparing the areas of the dark regions to those of the homozygous wild-type regions (highlighted by the brightest GFP labeling; Fig. 5d and e). We investigated the *H2Av^810^* mutation and heat shock-mediated Cas9 knockout mutation *H2Av^Cas9d^*, through the traditional mosaic analysis, which exhibited consistent clonal characteristics (Fig. 5d’ and e’). This confirms that using the *hs-Gal4*-induced Cas9 system to directly generate an FRT-combined mutant is feasible.

## Discussion

In recent years, gene regulatory technologies have rapidly developed, including RNAi and a series of CRISPR/Cas9-based genetic engineering techniques, which have revolutionized the field of genetic research. *D. melanogaster* has been a pivotal model organism for advancing our understanding of gene function, development, and disease. The CRISPR/Cas9 system, in particular, has emerged as a powerful tool for genome editing, enabling the knockout and knock-in of target genes, as well as other applications in chromosomal regulation. Recent advancements related to CRISPR/Cas9, such as the SAM (Synergistic Activation Mediator) system for gene activation (Lin et al. 2015; Han et al. 2022) and the KRAB (Krüppel-Associated Box) system for gene suppression (Parsi et al. 2017), have further expanded the utility of CRISPR/Cas9-based genetic technologies.

In *Drosophila*, several strategies have been developed to introduce Cas9 and *sgRNAs* for heritable mutations. Early approaches involved injecting Cas9 and *sgRNA* DNA templates (Gratz et al. 2014). Later, 2 groups reported that injecting in vitro-transcribed Cas9 mRNA and *sgRNA* into fly embryos improved mutagenesis efficiency (Bassett et al. 2013; Yu et al. 2013). However, in vitro RNA transcription is not only labor-intensive but also extremely costly. In addition, many injected embryos fail to develop to adulthood due to toxicity, necessitating the injection of hundreds of embryos per task. Alternatively, transgenic flies expressing Cas9 and *sgRNA* separately can be crossed to generate mutants (Kondo and Ueda 2013). Finally, several groups developed *nos-*Cas9 and *vas-*Cas9 docking fly strains that are highly suitable for gene editing by injecting only *sgRNA* constructs (Ren et al. 2013; Gratz et al. 2014; Sebo et al. 2014). However, these approaches often require complex genetic procedures and lack the flexibility for prescreening valuable genes and for high-throughput studies.

To address these limitations, we developed the versatile CRISPR/Cas9 binary expression system that integrates *UAS-Cas9* and *multiple*-*sgRNAs* into a single construct. This system simplifies experimental workflows by enabling direct phenotypic studies, clonal analysis, in vitro cellular assays, and the generation of stable mutants directly on an FRT background. By leveraging the Gal4/UAS binary system, we achieved flexible gene knockout and rough visible clonal analysis, significantly improving the methodological diversity and efficiency of studying gene defects and functions, particularly when suitable antibodies are unavailable. While these strategies have shown great promise, we acknowledge certain limitations, such as variability in knockout efficiency and spatial concordance, which depend on target gene properties and chromatin accessibility. Nevertheless, our system provides robust strategies for high-throughput genetic missions.

One of the key innovations in our study is the optimization of the CRISPR/Cas9 system using the *hs-Gal4* driver. Compared to continuously expressed Gal4 drivers, the heat shock system offers several advantages. First, it allows for precise temporal control of Cas9 expression, enabling gene knockout during early specific developmental stages. This is particularly beneficial for studying genes with stage-specific functions or those that are essential for early development. Second, it can produce in the early stages and produce larger clones with higher clonal induction efficiency, making it a powerful tool for clonal analysis. Third, its transient Cas9 expression minimizes the residual Cas9 activity in GSCs, reducing the risk of GSC loss. These features make the heat shock-optimized CRISPR/Cas9 system potential as a promising and effective genetic manipulation tool.

In conclusion, our CRISPR/Cas9 binary expression system represents a significant advancement in *Drosophila* genetics, offering a unified workflow for phenotypic studies, clonal analysis, and mutant generation. Challenges remain, such as further optimizing knockout efficiency and reducing off-target effects. Our approach provides a suite of useful tools for *Drosophila* genetic and developmental mechanism studies.

## Supplementary Material

jkaf236_Supplementary_Data

## Data Availability

The authors confirm that all data necessary to support the conclusions of this article are available within the article. Additionally, the plasmids, fly strains, and tools, and other detailed information, are available from the corresponding author upon request. Supplemental material available at G3 online.
