# Supplementary material for: Heat shock-optimized CRISPR/Cas9 system for visible clonal analysis and mutant generation in Drosophila
Source: G3 (Bethesda). 2025 Oct 7;15(12):jkaf236. doi: 10.1093/g3journal/jkaf236 (PMC12693565; doi:10.1093/g3journal/jkaf236)
Supplement: jkaf236_Supplementary_Data [file jkaf236_supplementary_data.zip › Document_S1_G3-2025-405858.docx]

**Supplementary Document 1**

1. **Primers for *G1*, *G2*, and *G3a/b* plasmid generation**

**Primers used for the identification of *G1*, *G2*, and *G3a/b* plasmids by bacterial PCR and Sanger sequencing:**

**pN**: TGGCCAGAGCTCTGCTAGCGA

**pM**: GCGACGAAAAGAACTGAAATAAAAC

**pO**:GCTAGCACTGAAATAAAACTAGTGG

**pQ**:CAAGCTTATCGCTAGCACTGAA

**pN-pM**: For identification and sequencing of the *dual-sgRNAs* insertion.

**PM-PO**: For identification and sequencing of an additional *dual-sgRNAs* inserted into the **SpeⅠ**site (upstream).

**PN-PQ**: For identification and sequencing of an additional *dual-sgRNAs* inserted into **NheⅠ**site(downstream).

1. **Detailed structure of the *dual-sgRNAs* PCR template plasmid**

**2.1 The PCR template plasmid used to generate the *dual-sgRNAs* fragment for insertion:**

**
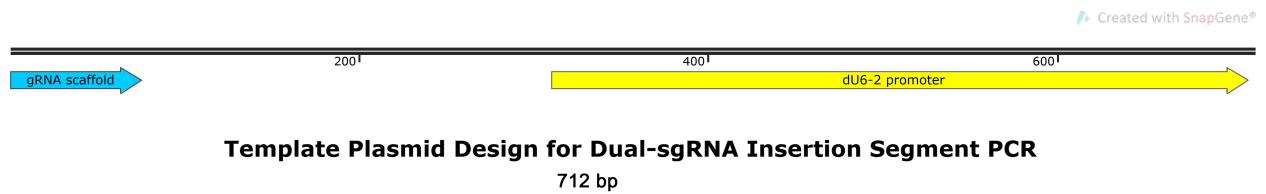
**

t**gttttagagctagaaatagcaagttaaaataaggctagtccgttatcaacttgaaaa**agtggcaccgagtcggtgcttttttatcgaattcctgcagcccgggggatccactagttctagagcggccgccaccgcggtggagctccagcttttgttccctttagtgagggttaatttcgagcttggcgtaatcatggtcatagctgtttcctgtgtgaaattgttatccgctcacaattccacacaacatacgagccggaagcataaagtgtaaagcctgctcgaggtcgacggtatcgataagcttgat**gttcgacttgcagcctgaaatacggcacgagtaggaaaagccgagtcaaatgccgaatgcagagtctcattacagcacaatcaactcaagaaaaactcgacacttttttaccatttgcacttaaatccttttttattcgttatgtatactttttttggtccctaaccaaaacaaaaccaaactctcttagtcgtgcctctatatttaaaactatcaatttattatagtcaataaatcgaactgtgttttcaacaaacgaacaataggacactttgattctaaaggaaattttgaaaatcttaagcagagggttcttaagaccatttgccaattcttataattctcaactgctctttcctgatgttgatcatttatataggtatgttttcctcaatacttc**g

**Note:** The plasmid was modified from ***dU6-BbsI-chiRNA* (Addgene** #**45946)**, and any completed *G1*, *G2*, or *G3* construct with the *dual-sgRNAs* inserted can also serve as a PCR template.

1. **Primers for *dual-sgRNAs* insertion fragments**

**3.1 Common primers for *dual-sgRNAs* cloning:**

**BbsⅠ-P5-Primer for *dual-sgRNAs* cloning PCR:**

TAGAAGACACCTTC***GNNNNNNNNNNNNNNNNNNN***GTTTTAGAGCTAGAAATAGCAAGT

**BbsⅠ-P3-Primer for *dual-sgRNAs* cloning PCR:**

TAGAAGACACCTTC***MMMMMMMMMMMMMMMMMMMC***GAAGTATTGAGGAAAACATACCTAT

**Note:** The **G** and **C** (shown above) are replaced with the appropriate base if the first nucleotide of the 20 nt *sgRNA* spacer sequence is not a G (for the U6 promoter) or C.

**Additional *dual-sgRNAs* amplification primers for generating 4X and 6X *multiple-sgRNAs* clusters:**

NheⅠ-DgRAmp-S: TTTGCTAGCACTGAAATAAAACTAGTGGATCCCC

NheⅠ-DgRAmp-A:TTTGCTAGCGATAAGCTTGATGTTCG

SpeⅠ-DgRAmp-S: TTTACTAGTACTGAAATAAATCTAGTGGATCCCC

SpeⅠ-DgRAmp-A:TTTACTAGTGATAAGCTTGATGTTCG

**Note:** For a 4x *sgRNAs* construct, the NheⅠ-DgRAmp primer pair is used first. To introduce the 5^th^ and 6^th^ ^sgRNAs^ using the SpeⅠ-DgRAmp primers, an internal SpeⅠ site (ACTAGT) must be mutated to TCTAGT in the first base. This mutation must also be incorporated into all subsequent NheⅠ-DgRAmp and SpeⅠ-DgRAmp primers.

**3.2 primer sequences for *dual-sgRNAs* targeting *GFP***

BbsⅠ-P5-Primer for *dual-sgRNAs* against *GFP* CDS upstream：

TAGAAGACACCTTC***GGTGGTGCAGATGAACTTCA***GTTTTAGAGCTAGAAATAGCAAGT

BbsⅠ-P3-Primer for *dual-sgRNAs* against *GFP* CDS downstream：

TAGAAGACACCTTC***CCCTGAGCAAAGACCCCAAC***GAAGTATTGAGGAAAACATACCTAT

**Note:** The following information shows only the 20 bp *sgRNA* spacer sequences. To target other genes, replace the bold and italic sequences in the *dual-gRNAs* primers for GFP with new spacer sequences.

**3.3 20nt spacer sequence in primers for targeting *Gal80^(ts)^* :**

*dual-gRNA-Gal80^(ts)^*-P5：******GATAGTGATAGCTATCCAAG******

*dual-gRNA-Gal80^(ts)^*-P3：******TTAGACCCGGGGTATGATGC******

**3.4 20nt spacer sequence in primers for targeting *H2av* :**

*dual-gRNA-H2av*-P5：******GGTAAAGCAGGCAAGGATTC******

*dual-gRNA-H2av*-P3：******TTCCGCTGCGGATCCTGCAC******

**3.5 20nt spacer sequence in primers for targeting *CG6236* :**

*dual-gRNA-CG6236*-P5：******GGATGTGATCTGCTCCTACA******

*dual-gRNA-CG6236*-P3：******GATCCCGGTCGTATATGCAC******

**3.6 20nt spacer sequence in primers for targeting *nonstop* :**

*dual-gRNA-nonstop*-P5：******GCACTATCAAAGCTATGTGA******

*dual-gRNA-nonstop*-P3：******GCACCTGCTTGAGCGATGCC******

**3.7 20nt spacer sequence in primers for targeting *dom* :**

*dual-gRNA-dom*-P5：******GTCTGGTTCAGAAGATGATG******

*dual-gRNA-dom*-P3：******CTCGAGGAGGCATTTCCTCC******

**3.8 20nt spacer sequence in primers for targeting *rbo* :**

*dual-gRNA-rbo*-P5：******GTGGATGCTGCTCAGCGCTC******

*dual-gRNA-rbo-*P3：******CGAGGGCGAGTTTGACGACC******

**3.9 20nt spacer sequence in primers for targeting *dlp* :**

*dual-gRNA-dlp*-P5：******GAAGCCTTTCGGAGATGTTC******

*dual-gRNA-dlp*-P3：******GAGCGCGACGCAGCGTCATC******

**3.10 20nt spacer sequence in primers for targeting *mad* :**

*dual-gRNA-mad*-P5：******GGGTGGAGTCACCCGATAAC******

*dual-gRNA-mad*-P3：******GTGCAGATGGATTTCAATCC******

**4. Fly lines for injection docking and mapping**

**4.1 Injection docking fly lines:**

Tsinghua Fly Center **(TB00016)**, for 2^nd^ chromosome docking:

*y[1] sc[1] v[1] p{y[+t7.7]}=nos-phiC31\int.NLS}X; P{y[+t7.7]=CaryP}attP40*

Or **(TB00018)**, for 3^rd^ chromosome docking:

*y[1] sc[1] v[1] p{y[+t7.7]}=nos-phiC31\int.NLS}X; P{y[+t7.7]=CaryP}attP2*

**4.2 Mapping fly tools:**

**(TB00023)**, for mapping inserts on the 2*^nd^* chromosome:

*y[1] sc[1] v[1]; wg[Gla-1] Bc[1]/CyO*

Or **(TB00139)**, for mapping inserts on the 3^rd^ chromosome:

*y[1] sc[1] v[1]; Dr[1] e[1]/TM3, Sb[1]*

**4.3 Genotypes of *G1* to *G3* genetic strains:**

*y[1] sc[1] v[1];* ***(G1,G2 or G3)*** */CyO*

Or  *y[1] sc[1] v[1];* ***(G1,G2 or G3)*** */TM3, Sb*

( Note: All generated lines are fertile and viable as homozygotes. )

**Supplementary Figure Legends**

**Fig. S1 Knockout efficiency of *H2Av* in S2 cells using the *G2* system.**

**(a-a’’) H2Av antibody staining confirmed that H2Av was almost completely eliminated in these cells, and both copies of the *H2Av* gene on homologous chromosomes were knocked out by the *G2* system. (b-b’’) In addition, the knockout effect in some cells failed or only one copy of *H2Av* gene was knocked out, making it weaker in staining intensity than the surrounding cells, the staining particles on the chromosome were significantly reduced. (c and c’) A representative field of view is shown. (d) Based on analysis of multiple cellular fields, the cumulative number of cells scored was n=30. The frequency of complete knockout cells was approximately 60%.**

**Fig. S2 The *G3b* system is functional for *H2Av* knockout in the trachea*.***

1. **A trachea cell clone, labeled by GFP expression via the *G3b* system. (a’) The expression of Flag-Cas9 is observed in this clone. (a’’) The efficiency knockout of the *H2Av* gene was indicated by antibody staining. The multi-channel merged image with DAPI nuclear staining is shown in (a’’’).**

**Fig. S3 Abnormal imaginal wing discs resulting from mad knockout using the *G3a* system.**

**The *mad* gene is critical for imaginal wing disc development. In these experiments, nearly 50% of larvae lacked visible wing discs by the late third instar larval stage. The remaining larvae often had abnormal discs that were easily observed (a-b’’). Clones (GFP-negative) at the disc boundary show a strong correlation with pMad-negative zones (a-a’’, b-b’’). Only a part of wing discs developed normally, as shown in Fig. 4h-h’’ in the main text ( less than 25% of individuals).**
