# Supplementary figures and images for: Heat shock-optimized CRISPR/Cas9 system for visible clonal analysis and mutant generation in Drosophila
Source: G3 (Bethesda). 2025 Oct 7;15(12):jkaf236. doi: 10.1093/g3journal/jkaf236 (PMC12693565; doi:10.1093/g3journal/jkaf236)

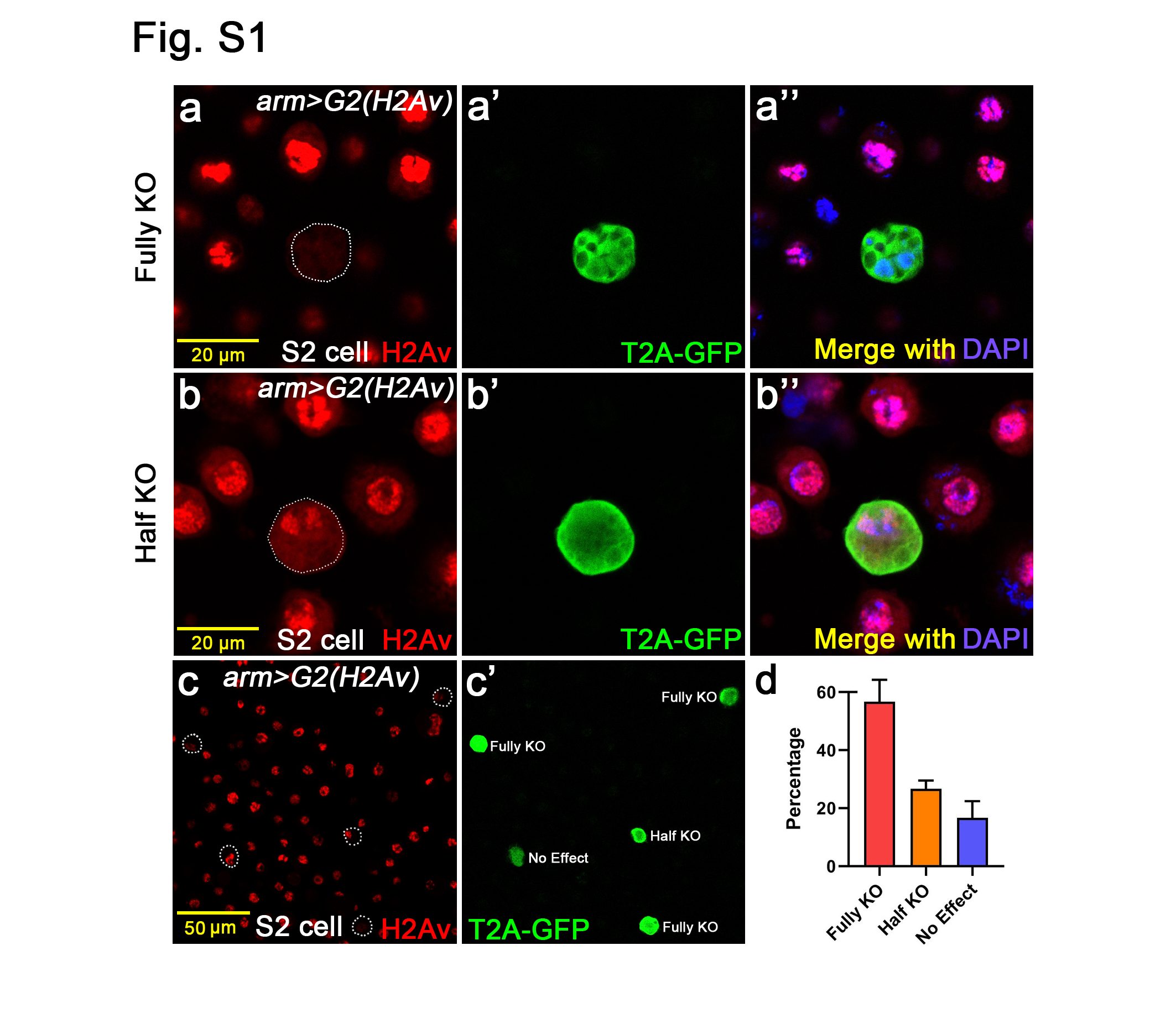

Supplement: jkaf236_Supplementary_Data [file jkaf236_supplementary_data.zip › Figure_S1_G3-2025-405858.png]

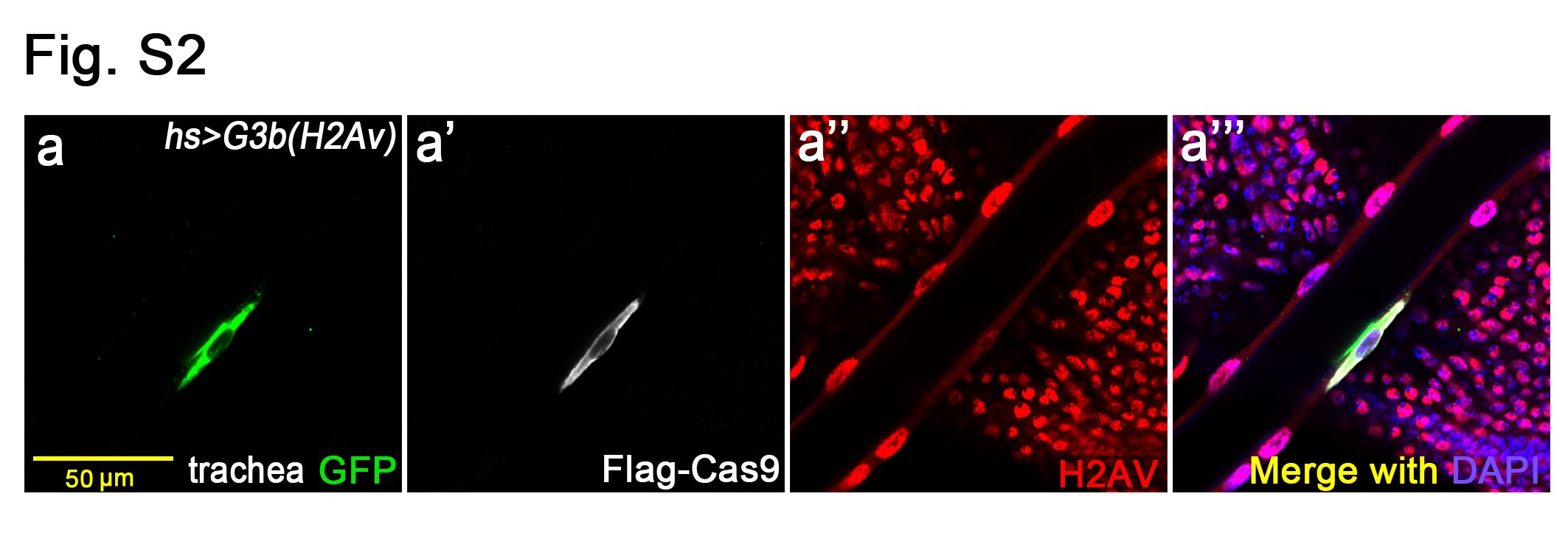

Supplement: jkaf236_Supplementary_Data [file jkaf236_supplementary_data.zip › Figure_S2_G3-2025-405858.png]

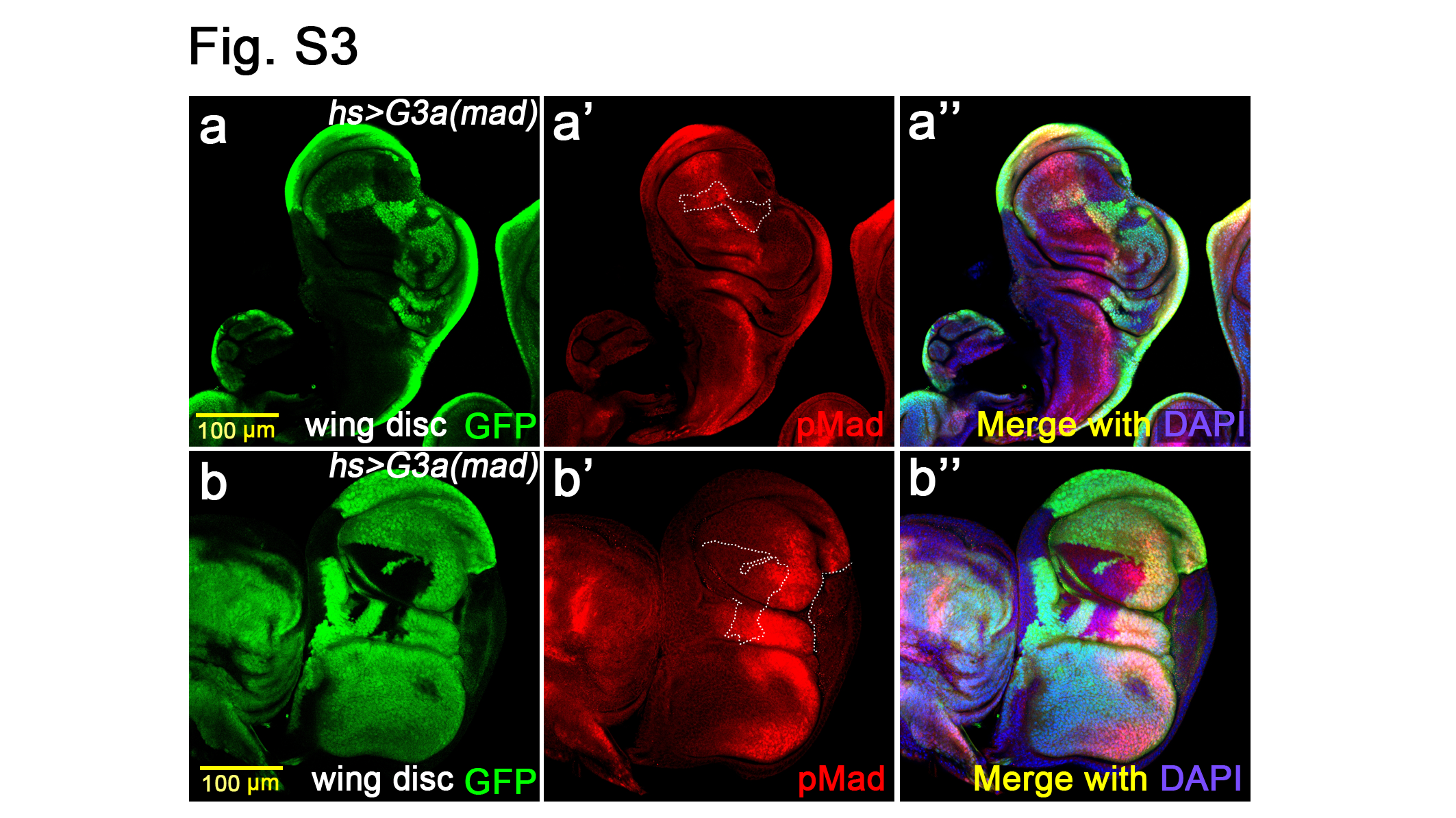

Supplement: jkaf236_Supplementary_Data [file jkaf236_supplementary_data.zip › Figure_S3_G3-2025-405858.png]
